# Prevalent peripheral arterial disease and inflammatory burden

**DOI:** 10.1186/s12877-016-0389-9

**Published:** 2016-12-09

**Authors:** Jane A. Cauley, Ahmed M. Kassem, Nancy E. Lane, Sara Thorson, for the Osteoporotic Fractures in Men (MrOS) Study Research Group

**Affiliations:** 1 Department of Epidemiology, Graduate School of Public Health, University of Pittsburgh, 130 DeSoto Street, A510 Crabtree Hall, Pittsburgh, PA 15261 USA; 2 University of California Davis Health System, 4625 2nd Avenue, Suite 2006, Sacramento, CA 95817 USA; 3 Family Medicine of Southwest, Peacehealth Southwest Medical Center, 400 NE Mother Joseph Place, Vancouver, WA 986664 USA

**Keywords:** Peripheral arterial disease, Peripheral vascular disease, Inflammation, Cytokine, Ankle-arm index, Ankle-brachial index, Smoking, Men

## Abstract

**Background:**

Strong evidence implicates inflammation in the development of atherosclerotic heart disease but less is known about peripheral arterial disease (PAD). Our objective was to test the hypothesis that a composite index of inflammatory burden is associated with PAD.

**Methods:**

Cross-sectional analysis of a randomly-selected group of 903 community-dwelling men in the MrOS cohort recruited between 2000 and 2002. Using blood samples, we measured seven cytokines and related these levels to prevalent PAD (ankle-brachial index (ABI) <0.9) both individually and as part of an “inflammatory burden score” (a composite sum of the number of pro-inflammatory cytokines in the highest quartile).

**Results:**

Overall, 6.75% of men had ABI <0.9. The odds of prevalent PAD were higher in men with the highest quartile (Q4) levels of interleukin-6 multivariable (MV) adjusted (odds ratio (OR) =3.95 (95% CI, 1.4–11.3), tumor necrosis factor alpha OR = 4.44 (95% confidence interval (CI), 1.5–12.8), and C-reactive protein OR = 3.63 (95% CI, 1.4–9.4) compared to men in Q1. The magnitude of the association of these cytokines with PAD was similar to the effect of being 10 years older, OR = 2.41 (95% CI, 1.16–3.7). These significant effects persisted after additional MV adjustment for smoking except for CRP. Men with the highest inflammatory burden score (≥3) had 3.6 (95% CI, 1.5–8.7) increased odds of PAD, *p* trend = 0.03. After smoking adjustment the linear trend was borderline statistically significant (*p* trend = 0.10).

**Conclusion:**

Inflammatory burden is associated with prevalent PAD, an association similar to aging 10 years. The inflammatory effects of smoking contributes to the underlying association between inflammation and PAD.

## Background

Peripheral arterial disease (PAD) affects more than 8 million Americans and its prevalence is likely to increase as the population ages [1]. Not only is the disease linked to significantly impaired physical function and quality of life [2–4] but considerable evidence supports a higher risk of mortality in patients with PAD [5, 6], particularly from cardiovascular causes [7, 8]. The ankle brachial index (ABI), which is the ratio of systolic pressure at posterior tibial and/or dorsalis pedis arteries divided by the brachial systolic blood pressure, is commonly used to measure PAD in the legs. Interestingly, incorporation of the ABI [6] into risk stratification tools such as Framingham Risk Score (includes age, total and high density lipoprotein cholesterol, blood pressure, diabetes and smoking) was found to nearly double the accuracy of 10-year predictions of total mortality, cardiovascular mortality, and major coronary events [6].

A number of recent studies have found pro-inflammatory cytokines to be strongly linked to prevalent PAD [9–11] as well as its severity [12, 13] even after adjusting for traditional cardiovascular risk factors [10, 13–15]. While the mechanisms remain unknown, certain pro-inflammatory cytokines, such as interleukin-6 (IL-6) and tumor-necrosis factor-α (TNFα), TNF soluble receptor-II (TNFαSRII), appear to be associated with PAD independent of one another [15]. Some inflammatory markers show different relationships with PAD in the context of disease, as is the case with C-reactive protein (CRP) and diabetes [16].

The exact pathophysiology of PAD remains unclear, but inflammation appears to be involved. Previous studies relating inflammatory markers to PAD had small sample sizes or included only a few inflammatory cytokines. Using data collected in the Osteoporotic Fractures in Men Study (MrOS), we evaluated the relation of 7 inflammatory markers - CRP, IL-6, IL-6 soluble receptor (IL-6SR), TNFα, TNFαSRI, and TNFαSRII as well as interleukin-10 (IL-10, anti-inflammatory), to prevalent PAD in older men and identified characteristics common to those with the highest cytokine inflammatory burden.

## Methods

### Participants

In 2000–2002, 5994 men enrolled in the Osteoporotic Fractures in Men Study (MrOS), a longitudinal cohort study designed to determine risk factors for osteoporosis, fracture and falls. Men were recruited at six US academic clinical centers primarily through mass mailings targeted to age eligible men. The MrOS study is described in more detail in other publications [17, 18]. Briefly, all men were age ≥ age 65, able to walk independently and did not report bilateral hip replacements; 35% of men were age 65–59 and 11% age 80 or older at baseline. The study was approved by the Institutional Review Boards at each institution. All participants provided written informed consent.

The current analysis was limited to a cohort of 1,530 randomly-selected participants. Men with at least 5, 1-mL aliquots of archived, unthawed serum were eligible for inclusion. Of these, 980 participants had cytokine data. These men were from a randomly selected subcohort of MrOS men who had inflammatory markers measured as part of a case-cohort study of inflammation and fracture [19]. In order to assign an inflammatory burden score (see below), we excluded 46 participants with missing cytokine data. We further excluded 31 participants with missing ABI values and additional 25 participants with ABI > =1.4, rendering an analytic sample of 878.

### Measurements at baseline visit

#### Biochemical measurements

Fasting morning blood samples were obtained at the baseline visit and were processed and stored at -120C until assay. All cytokine assays were performed at the Laboratory for Cytokine Biochemistry (LCBR), University of Vermont, under the direction of Dr. Russell Tracy.

IL-6 was measured using a high sensitivity ELISA from R&D Systems (Minneapolis, MN) employing a quantitative sandwich enzyme immunoassay technique. The assay range is 0.16–12.0 pg/mL with inter-assay coefficients of variability (CVs) ranging from 6.11–8.47%. Expected values for IL-6 in normal, healthy individuals are <10 pg/mL.

IL-6sR, TNFαSRI and TNFαSRII were measured using an ELISA from R&D Systems (Minneapolis, MN). A monoclonal antibody specific for each cytokine receptor was coated on the assay plate and a polyclonal anti- cytokine receptor antibody used as the sandwich antibody; the amount of cytokine receptor was then determined by colorimetric reaction. The assay range for IL-6sR was 3120–200,000 pg/mL. The manufacturer normal range for IL-6 is approximately 15,000-46,000 pg/mL with inter-assay CVs ranging from 4.68–8.83%. The assay range for TNFα SRI and SRII is 78–6000 pg/mL with inter-assay CVs ranging from 5.42–8.59% for TNFαSRI and 2.87–3.54% for TNFαSRII.

IL-10 and TNFα were measured using the Human Serum CVD3 Multiplex Kit from Millipore Corp. (Billerica, MA), using flow cytometry on the Bio-Rad Bioplex 200 Luminex instrument. The assay range for IL-10 is 0.13-2000 pg/mL with inter-assay CVs ranging from 4.94–10.66%. The TNFα assay range is 0.13-2000 pg/mL with inter-assay CVs ranging from 4.93–9.13%.

CRP was measured using the BNII nephelometer from Dade Behring utilizing a particle enhanced immunonepholometric assay. The assay range is 0.16–1100 ug/mL. Expected values for CRP in normal, healthy individuals are ≤ 3 ug/mL. Inter-assay CVs ranged from 1.52–3.68%.

Participants were assigned a pro-inflammatory burden score –a composite variable that was the sum of the number of pro-inflammatory cytokines (IL-6, IL-6 SR, TNFα, TNFαSRI, TNFαSRII, CRP) in the highest quartile. Of the 878 men in our analytic sample, 851(97%) had data on all 6 inflammatory markers and an additional 26 men had data on 5 cytokines. Based on the pro-inflammatory burden score, participants were classified into 4 groups according to the number of pro-inflammatory cytokines in the highest quartile as follows: 0, 1, 2, and 3–6 (≥3). Other published studies have found relationships between inflammatory burden scores and hip fracture [20].

### Outcome measurements

The systolic blood pressure in the right and left posterior tibial artery and the right brachial artery were measured twice after the subjects were supine for at least 5 min. The pulses were detected by using a hand held 8 MHz doppler. The resting ABI was calculated in each leg as the average of the 2 measures of the posterior tibial pressure divided by the average of the two brachial measures. The lower of the right and left index was used as a measure of lower extremity PAD [21]. Participants with an ABI < 0.90 were classified as having PAD, while those with an ABI ≥0.90 were considered free of disease [6].

### Covariates

Height was measured by Harpenden stadiometer; weight, by digital or balance beam scale. Body mass index (BMI) was calculated as weight in kilograms divided by height in meters squared. Biomarkers included systolic blood pressure (average of two measurements in the arm), fasting blood glucose, total cholesterol and HDL. Walking speed was determined by time to complete a 6-m course at the participant’s usual walking speed [18].

A self-administered questionnaire included demographics, education, self-rated health status, medical history, tobacco use (pack-years and current smoking/past smoking status), and alcohol consumption. Participants were asked to bring all prescription medications they had been taking for at least 1 month to the clinic visit. All medications were entered into an electronic database, verified by pill bottle examination, and each medication was matched to its ingredients based on the Iowa Drug Information Service (IDIS) Drug Vocabulary (College of Pharmacy, University of Iowa, Iowa City, IA) [22]. Participants were classified as diabetic by self-report, a fasting blood glucose (FBG) ≥126 mg/dL, or if they had prescriptions for hypoglycemic agents. Pre-diabetes was defined as FBG ≥100 mg/dL and <126 mg/dL, while normoglycemia was defined as blood glucose <100 mg/dL. Physical activity was quantified using the Physical Activity Scale for the Elderly (PASE) [23].

### Statistical analysis

We calculated descriptive statistics for all variables and tabulated characteristics of participants by level of pro-inflammatory burden score testing for trend. Next, we compared participants with and without PAD. For this, we used chi-squared test (or Fisher's exact test) for categorical variables, two-sample *t* test for normally distributed continuous data and Wilcoxon-Mann–Whitney test for skewed continuous data. Median tests were used for the cytokines variables because of skewed distributions. Then, a series of crude, age-adjusted and multivariable-adjusted logistic regression models of the relationship between each cytokine and PAD were fit. We modeled quartiles of cytokines as dummy variables (1–4) with quartile 1 as the referent. For the inflammatory burden logistic regressions, we also used dummy variables to account for the number of “high” inflammatory cytokines (0, 1, 2, ≥3). The multivariable models were adjusted for variables that were significantly different between men with and without PAD and variables that are related to both PAD and inflammation. All statistical analyses were conducted with Stata version 13.1 (StataCorp LP, College Station, TX, USA).

## Results

Descriptive characteristics of study participants by inflammatory burden score (0 to ≥3) are shown in Table 1. The largest percentage of participants (35%) had a score of 0, followed by a score of 1 (26%), 2 (15%) and ≥3 (24%). With increasing inflammatory burden score, the prevalence of PAD as measured by ABI increased. Men with the highest inflammatory burden also tended to be older and were less likely to rate their health status as good or excellent. Fasting blood glucose tended to increase but total cholesterol and HDL tended to decrease with increasing inflammatory burden. Men with three or more cytokines measured in the highest quartile had a higher prevalence of multiple medical conditions including history of myocardial infarction, hypertension, chronic obstructive pulmonary disease (COPD), congestive heart failure (CHF) and diabetes when compared to those with a score of 0 or 1. A higher proportion of participants with the highest inflammatory burden were unable to walk faster than 0.8 m/s. There was no difference in use of aspirin or non-steroidal anti-inflammatory drugs (NSAIDs) across inflammatory burden but use of ACE inhibitors, loop diuretics and antidepressants was greatest among men with the greatest inflammatory burden. On average, men with the highest inflammatory burden also tended to drink less alcohol and have a greater number of smoking pack-years.

**Table 1:** Characteristics of participants according to number of pro-inflammatory cytokines in the highest quartile

Characteristics	Total *N* = 878	Pro-inflammatory Burden Score^a^				*P* for trend
		0 *N* = 302	1 *N* = 230	2 *N* = 131	≥3 *N* = 215	
PAD, ABI <0.90, n (%)	61 (6.95)	12 (3.97)	14 (6.09)	5 (3.82)	30 (13.95)	<0.001
Demographics						
Age, years, mean ± SD	73.71 ± 5.87	72.82 ± 5.39	72.44 ± 5.13	74.22 ± 6.10	75.99 ± 5.87	<0.001
Non-white race, n (%)	67 (7.63)	29 (9.60)	21 (9.13)	7 (5.34)	10 (4.65)	0.019
High school or less, n (%)	217 (24.72)	56 (18.54)	59 (25.65)	40 (30.53)	62 (28.84)	0.003
Health status						
Self-rated health status, good/excellent, n (%)	753 (85.86)	275 (91.06)	201 (87.39)	111 (85.38)	166 (77.21)	<0.001
Body Mass Index (BMI), mean ± SD	27.32 ± 3.56	27.22 ± 3.17	27.06 ± 3.72	27.39 ± 3.57	27.69 ± 3.86	0.146
Systolic blood pressure (mmHG), mean ± SD	139.09 ± 18.92	138.94 ± 18.44	138.60 ± 18.59	138.58 ± 18.06	140.13 ± 20.47	0.555
Fasting blood glucose (mg/dL), mean ± SD	106.68 ± 24.90	105.20 ± 21.53	104.22 ± 22.69	109.37 ± 32.89	109.75 ± 25.53	0.027
Total cholesterol (mg/dL), mean ± SD	194.58 ± 32.84	196.43 ± 32.52	196.78 ± 32.69	196.30 ± 36.28	188.59 ± 30.62	0.021
HDL (mg/dL), mean ± SD	48.96 ± 14.65	51.05 ± 14.39	49.85 ± 15.46	48.35 ± 13.75	45.46 ± 14.11	<0.001
Medical conditions						
Myocardial infarction, n (%)	139 (15.83)	41 (13.58)	29 (12.61)	20 (15.27)	49 (22.79)	0.005
Hypertension, n (%)	383 (43.62)	122 (40.40)	84 (36.52)	56 (42.75)	121 (56.28)	<0.001
Congestive heart failure, n (%)	36 (4.10)	7 (2.32)	5 (2.17)	4 (3.05)	20 (9.30)	<0.001
Chronic obstructive pulmonary disease, n (%)	97 (11.05)	17 (5.63)	26 (11.30)	17 (12.98)	37 (17.21)	<0.001
Diabetes status						0.002
Diabetes, n (%)	142 (16.69)	39 (13.36)	25 (11.21)	24 (18.75)	54 (25.96)	
Pre-diabetes, n (%)	310 (36.43)	114 (39.04)	79 (35.43)	44 (34.38)	73 (35.10)	
Medication use						
NSAID, n (%)	130 (15.51)	44 (15.28)	26 (11.82)	19 (15.45)	41 (19.81)	0.137
Aspirin, n (%)	284 (33.89)	97 (33.68)	73 (33.18)	32 (26.02)	82 (39.61)	0.366
Cox-II inhibitor, n (%)	63 (7.52)	20 (6.94)	11 (5.00)	10 (8.13)	22 (10.63)	0.090
ACE inhibitors, n (%)	166 (19.81)	53 (18.40)	30 (13.64)	24 (19.51)	59 (28.50)	0.004
Loop diuretics, n (%)	38 (4.53)	1 (0.35)	6 (2.73)	4 (3.25)	27 (13.04)	<0.001
Antidepressants, n (%)	55 (6.65)	15 (5.26)	13 (6.07)	3 (2.46)	24 (11.65)	0.022
Physical performance						
Physical Activity Scale for the Elderly score, mean ± SD	149.92 ± 69.95	155.79 ± 66.48	158.76 ± 67.01	147.00 ± 65.49	134.02 ± 77.68	<0.001
Walking speed <0.8 meters/seconds, n (%)	28 (3.19)	7 (2.32)	3 (1.30)	3 (2.29)	15 (6.98)	0.004
Average number of drinks/week, mean ± SD	4.52 ± 6.90	5.23 ± 7.20	4.78 ± 7.25	3.61 ± 5.82	3.82 ± 6.60	0.001
Smoking status						0.010
Current smoker, n (%)	33 (3.76)	6 (1.99)	7 (3.04)	7 (5.34)	13 (6.05)	
Past smoker, n (%)	514 (58.54)	172 (56.95)	134 (58.26)	74 (56.49)	134 (62.33)	
Smoking pack-years, mean ± SD	18.97 ± 25.71	16.09 ± 23.86	18.09 ± 24.74	18.04 ± 22.40	24.53 ± 30.07	0.003

^a^Composite variable summing number of pro-inflammatory cytokines (IL-6, IL-6 SR, TNFα, TNFαSRI, TNFαSRII, CRP) in the highest quartile

Men with PAD (6.75%) had higher median levels of the pro-inflammatory cytokines IL-6, IL-10, TNFα, TNFαSRI, TNFαSRII, and CRP; they were almost twice as likely to have a CRP level above the clinical cutoff of 3ug/mL (*p* < 0.05), Table 2. They were also older, and less likely to report good/excellent health status, had higher rates of hypertension, and CHF. Systolic blood pressure and fasting glucose were significantly higher in men with PAD. BMI was lower in the PAD group. There was no difference in NSAIDs, aspirin use, Cox inhibitors or alcohol use by PAD status. Men with ABI <0.9 were more likely to report use of ACE inhibitors, loop diuretics and antidepressants (*p* = 0.08).

**Table 2:** Characteristics of men with and without peripheral arterial disease

Characteristics	Total *N* = 878	Men with PAD (ABI <0.90) *N* = 61	Men without PAD (ABI ≥0.90) *N* = 817	*p*-value^a^
Cytokines				
IL-6 pg/ml, median (interquartile range)	2.40 (1.84)	3.38 (2.16)	2.34 (1.71)	<0.001
IL-6 SR pg/ml, median (interquartile range)	48636 (18103)	50655 (19051)	48494 (18064)	0.232
IL-10 pg/ml, median (interquartile range)	8.98 (6.94)	10.62 (5.69)	8.87 (6.86)	0.012
TNFα pg/ml, median (interquartile range)	3.95 (2.43)	4.55 (2.24)	3.91 (2.41)	0.032
TNFαSRI pg/ml, median (interquartile range)	1918.50 (586.90)	2210.50 (853.10)	1900.70 (570.10)	0.002
TNFαSRII pg/ml, median (interquartile range)	3499.80 (952.10)	3856.85 (1202.90)	3482.10 (922.70)	0.029
CRP ug/ml, median (interquartile range)	1.40 (2.12)	2.24 (3.94)	1.37 (2.08)	0.042
CRP >3 ug/mL, n (%)	209 (23.94)	24 (40.68)	185 (22.73)	0.002
Demographics				
Age, years, mean ± SD	73.71 ± 5.87	76.70 ± 5.99	73.48 ± 5.99	<0.001
None-white race, n (%)	67 (7.63)	4 (6.56)	63 (7.71)	1.000
High school or less, n (%)	217 (24.72)	19 (31.15)	198 (24.24)	0.227
Health status				
Self-rated health status, good/excellent, n (%)	753 (85.86)	44 (72.13)	709 (86.89)	0.001
Body Mass Index (BMI), mean ± SD	27.32 ± 3.56	26.05 ± 3.25	27.42 ± 3.56	0.004
Systolic blood pressure (mmHG), mean ± SD	139.09 ± 18.92	148.21 ± 24.13	138.41 ± 18.31	<0.001
Fasting blood glucose (mg/dL), mean ± SD	106.68 ± 24.90	117.80 ± 41.47	105.83 ± 22.97	0.039
Total cholesterol (mg/dL), mean ± SD	194.58 ± 32.84	191.44 ± 32.46	194.82 ± 32.87	0.442
HDL (mg/dL), mean ± SD	48.96 ± 14.65	48.32 ± 15.85	49.01 ± 14.57	0.435
Medical conditions				
Myocardial infarction, n (%)	139 (15.83)	9 (14.75)	130 (15.91)	0.811
Hypertension, n (%)	383 (43.62)	39 (63.93)	344 (42.11)	0.001
Congestive heart failure, n (%)	36 (4.10)	7 (11.48)	29 (3.55)	0.003
Chronic obstructive pulmonary disease, n (%)	97 (11.05)	8 (13.11)	89 (10.89)	0.593
Diabetes status				0.071
Diabetes, n (%)	142 (16.69)	16 (26.67)	126 (15.93)	
Pre-diabetes, n (%)	310 (36.43)	22 (36.67)	288 (36.41)	
Medication use				
NSAID, n (%)	130 (15.51)	10 (17.54)	120 (15.36)	0.661
Aspirin, n (%)	284 (33.89)	20 (35.09)	264 (33.80)	0.843
Cox-II inhibitor, n (%)	63 (7.52)	1 (1.75)	62 (7.94)	0.115
ACE inhibitors, n (%)	166 (19.81)	18 (31.58)	148 (18.95)	0.021
Loop diuretics, n (%)	38 (4.53)	8 (14.04)	30 (3.84)	<0.001
Antidepressants, n (%)	55 (6.65)	7 (12.28)	48 (6.23)	0.077
Physical performance				
Physical Activity Scale for the Elderly (PASE) score, mean ± SD	149.92 ± 69.95	130.25 ± 79.94	151.39 ± 68.98	0.008
Walking speed <0.8 meters/seconds, n (%)	28 (3.19)	6 (9.84)	22 (2.69)	0.002
Average number of drinks/week, mean ± SD	4.52 ± 6.90	4.77 ± 7.79	4.51 ± 6.84	0.275
Smoking status				<0.001
Current smoker, n (%)	33 (3.76)	8 (13.11)	25 (3.06)	
Past smoker, n (%)	514 (58.54)	40 (65.57)	474 (58.02)	
Smoking pack-years, mean ± SD	18.97 ± 25.71	33.64 ± 32.44	17.87 ± 24.81	<0.001

^a^Based on median test for cytokines variables, two-sample *t* test (or Wilcoxon-Mann–Whitney test) for other continuous variables and chi-squared test (or Fisher's exact test) for categorical variables

Men with PAD were 4.4 times more likely to be currently smoking that those without the disease; they also had nearly twice the number of smoking pack-years.

Trends between quartile levels of various cytokines and prevalent PAD are shown in Table 3. Current smoking status was added separately to multivariate adjustment because it was found to have an important influence on odds ratios.

**Table 3:** Association between cytokines and prevalent peripheral arterial disease (ABI <0.90)

		Second quarter (25th - 50th percentile)	Third quarter (50th -75th percentile)	Fourth quarter (75th - 100th percentile)	*P* for trend
Cytokine	Model	Odds Ratio, 95% Confidence Interval^a^			
IL-6	Crude model	1.94 (0.65, 5.87)	3.63 (1.31, 10.01)	6.00 (2.27, 15.91)	<0.001
	Age-adjusted model	1.71 (0.57, 5.12)	3.13 (1.12, 8.71)	4.91 (1.83, 13.17)	0.001
	Multivariate-adjusted model^b^	1.37 (0.42, 4.46)	3.46 (1.19, 10.11)	3.95 (1.38, 11.26)	0.003
	Multivariate-adjusted model + smoking status	1.32 (0.40, 4.31)	2.88 (0.98, 8.49)	2.90 (0.99, 8.52)	0.020
	Multivariate-adjusted model + CVD risk factors^c^	1.17 (0.34, 3.98)	3.39 (1.13,10.18)	3.57 (1.21, 10.50)	0.005
IL-6 SR	Crude model	0.95 (0.42, 2.13)	1.11 (0.51, 2.43)	1.71 (0.84, 3.48)	0.129
	Age-adjusted model	0.93 (0.41, 2.10)	1.07 (0.49, 2.35)	1.61 (0.78, 3.31)	0.180
	Multivariate-adjusted model^b^	1.16 (0.46, 2.89)	1.12 (0.47, 2.71)	1.65 (0.73, 3.74)	0.270
	Multivariate-adjusted model + smoking status	1.24 (0.49, 3.18)	1.31 (0.52, 3.28)	1.85 (0.79, 4.36)	0.170
	Multivariate-adjusted model + CVD risk factors^c^	1.12 (0.44, 2.89)	1.03 (0.41, 2.59)	1.86 (0.79, 4.34)	0.204
IL-10	Crude model	1.30 (0.53, 3.14)	2.73 (1.23, 6.05)	1.89 (0.82, 4.34)	0.045
	Age-adjusted model	1.27 (0.52, 3.11)	2.55 (1.14, 5.68)	1.63 (0.70, 3.79)	0.107
	Multivariate-adjusted model^b^	1.48 (0.56, 3.93)	2.02 (0.81, 5.04)	1.57 (0.62, 3.96)	0.263
	Multivariate-adjusted model + smoking status	1.52 (0.56, 4.13)	2.13 (0.83, 5.44)	1.75 (0.68, 4.53)	0.184
	Multivariate-adjusted model + CVD risk factors^c^	1.74 (0.63, 4.81)	2.52 (0.96, 6.57)	1.89 (0.72, 4.95)	0.139
TNFα	Crude model	2.33 (0.93, 5.83)	2.52 (1.02, 6.21)	3.24 (1.35, 7.79)	0.010
	Age-adjusted model	2.43 (0.97, 6.13)	2.54 (1.02, 6.28)	3.09 (1.27, 7.47)	0.015
	Multivariate-adjusted model^b^	3.02 (0.99, 9.18)	3.69 (1.26, 10.78)	4.44 (1.54, 12.80)	0.005
	Multivariate-adjusted model + smoking status	2.95 (0.96, 9.06)	3.40 (1.15, 10.08)	3.95 (1.35, 11.58)	0.012
	Multivariate-adjusted model + CVD risk factors^c^	3.37 (1.05, 10.83)	4.13 (1.35, 12.67)	5.34 (1.77, 16.16)	0.003
TNFαSRI	Crude model	1.15 (0.47, 2.84)	1.54 (0.65, 3.64)	3.34 (1.53, 7.29)	0.002
	Age-adjusted model	1.01 (0.41, 2.51)	1.25 (0.52, 2.99)	2.24 (0.97, 5.15)	0.051
	Multivariate-adjusted model^b^	1.24 (0.46, 3.34)	1.28 (0.48, 3.42)	2.26 (0.89, 5.75)	0.104
	Multivariate-adjusted model _+_ smoking status	1.16 (0.42, 3.16)	1.07 (0.39, 2.89)	1.80 (0.69, 4.69)	0.285
	Multivariate-adjusted model + CVD risk factors^c^	1.43 (0.50, 4.13)	1.59 (0.56, 4.52)	3.20 (1.15, 8.85)	0.029
TNFαSRII	Crude model	1.83 (0.72, 4.68)	1.95 (0.77, 4.94)	3.74 (1.57, 8.88)	0.004
	Age-adjusted model	1.64 (0.64, 4.21)	1.64 (0.64, 4.20)	2.65 (1.08, 6.54)	0.042
	Multivariate-adjusted model^a^	1.79 (0.63, 5.06)	1.70 (0.60, 4.83)	2.50 (0.91, 6.85)	0.095
	Multivariate-adjusted model ^+^ smoking status	1.50 (0.52, 4.33)	1.46 (0.50, 4.20)	2.11 (0.75, 5.91)	0.181
	Multivariate-adjusted model + CVD risk factors^c^	2.23 (0.72, 6.93)	2.66 (0.85, 8.32)	3.81 (1.25, 11.63)	0.019
CRP	Crude model	1.78 (0.73, 4.32)	1.53 (0.61, 3.82)	3.37 (1.48, 7.64)	0.008
	Age-adjusted model	1.92 (0.78, 4.71)	1.66 (0.66, 4.18)	3.43 (1.50, 7.85)	0.008
	Multivariate-adjusted model^b^	2.61 (0.95, 7.15)	1.88 (0.66, 5.36)	3.63 (1.41, 9.36)	0.020
	Multivariate-adjusted model + smoking status	2.41 (0.87, 6.66)	1.52 (0.52, 4.44)	2.80 (1.06, 7.37)	0.092
	Multivariate-adjusted model + CVD risk factors^c^	2.02 (0.71, 5.73)	1.62 (0.56, 4.71)	3.24 (1.23, 8.55)	0.035

^a^Reference group = lowest quartile

^b^Multivariate-adjusted models were adjusted for age, MI, hypertension, CHF, COPD, diabetes status, NSAID, Aspirin, cox-II inhibitor, ACE inhibitors, loop diuretics, antidepressants, self-rated health status, BMI, PASE score and walking speed

^c^CVD risk factors included systolic blood pressure, fasting blood glucose, total cholesterol and HDL

There were higher odds of prevalent PAD among participants with increasing levels of IL-6, TNFα, and CRP. These trends remained statistically significant (*p* <0.05) in MV models, except in the case of CRP, which was attenuated after multivariate plus smoking status adjustment. For IL-6, in MV models, the odds ratios ranged from 1.37 (quartile 2) to 3.95 (quartile 4), *p* trend = 0.003; for TNFα, 3.02 (quartile 2) to 4.44 (quartile 4), *p* trend = 0.05; and for CRP, 2.61 (quartile 2) to 3.63 (quartile 4), *p* trend = 0.02. These increased odds of prevalent PAD were similar to aging 10 years (OR per ten year increase in age = 2.41 (1.57 - 3.70). A positive trend between TNFαSRI and TNFαSRII was found with PAD for crude and age-adjusted models; however, this was no longer statistically significant after multivariate adjustment or smoking adjustment. The association with IL-6 SR and Il-10 with PAD were no longer significant in the age adjusted models. Adjustment for cardiovascular risk factors had little effect on the association between inflammatory burden and PAD.

Men with CRP >3ug/mL were more likely to have prevalent PAD than those with a lower level, MV models, OR = 2.0, (95% CI, 1.06–3.79). Nevertheless, this association was no longer significant after adjusting for pack-years or smoking status (Table 4). Participants with an inflammatory burden score ≥3 (Table 5) were 3.6 times more likely to have PAD compared to those with a score of 0, OR = 3.59 (95% CI, 1.48–8.71). This trend was attenuated slightly after adjustment for smoking, (p trend = 0.09).

**Table 4:** Association between CRP >3 ug/mL and prevalent peripheral arterial disease (ABI <0.90)

Model	CRP >3 ug/mL
	Odds Ratio (95% Confidence Interval)^a^
Crude model	2.33 (1.35, 4.02)
Age-adjusted model	2.24 (1.29, 3.90)
Conditions-adjusted model^b^	2.23 (1.26, 3.96)
Medications-adjusted model^c^	2.20 (1.23, 3.96)
Multivariate-adjusted model^d^	2.00 (1.06, 3.79)
Multivariate-adjusted model + smoking status	1.69 (0.88, 3.25)
Multivariate-adjusted model + smoking pack-years	1.69 (0.87, 3.26)
Multivariate-adjusted model + CVD risk factors^e^	2.02 (1.04, 3.92)

^a^Reference group = CRP ≤3 ug/mL

^b^Conditions-adjusted model was adjusted for MI, hypertension, CHF, COPD, diabetes status

^c^Medications-adjusted model was adjusted for NSAID, Aspirin, cox-II inhibitor, ACE inhibitors, loop diuretics, antidepressants

^d^Multivariate-adjusted models were adjusted for age, MI, hypertension, CHF, COPD, diabetes status, NSAID, Aspirin, cox-II inhibitor, ACE inhibitors, loop diuretics, antidepressants, self-rated health status, BMI, PASE score, walking speed

^e^CVD risk factors included systolic blood pressure, fasting blood glucose, total cholesterol and HDL

**Table 5:** Association of pro-inflammatory burden scores with prevalent peripheral arterial disease

Model	Pro-inflammatory burden score			
	Odds Ratio (95% Confidence Interval)^a^			
	1	2	≥3	*P* for trend
Crude model	1.57 (0.71, 3.45)	0.96 (0.33, 2.78)	3.92 (1.96, 7.85)	0.002
Age-adjusted model	1.62 (0.73, 3.59)	0.85 (0.29, 2.48)	3.11 (1.52, 6.35)	0.025
Conditions-adjusted model^b^	1.79 (0.79, 4.06)	1.01 (0.34, 2.98)	3.65 (1.75, 7.63)	0.008
Medications-adjusted model^c^	2.13 (0.90, 5.06)	1.30 (0.42, 3.98)	4.29 (1.93, 9.54)	0.004
Multivariate-adjusted model^d^	2.40 (0.95, 6.07)	1.27 (0.39, 4.15)	3.59 (1.48, 8.71)	0.031
Multivariate-adjusted model + smoking status	2.25 (0.87, 5.78)	1.15 (0.35, 3.79)	2.88 (1.17, 7.13)	0.097
Multivariate-adjusted model + smoking pack-years	2.42 (0.93, 6.27)	1.28 (0.38, 4.25)	3.29 (1.33, 8.15)	0.052
Multivariate-adjusted model + CVD risk factors^e^	2.87 (1.06, 7.79)	1.31 (0.37, 4.67)	4.88 (1.86, 12.79)	0.013

^a^Reference group = pro-inflammatory burden score of zero

^b^Conditions-adjusted model was adjusted for MI, hypertension, CHF, COPD, diabetes status

^c^Medications-adjusted model was adjusted for NSAID, Aspirin, cox-II inhibitor, ACE inhibitors, loop diuretics, antidepressants

^d^Multivariate-adjusted models were adjusted for age, MI, hypertension, CHF, COPD, diabetes status, NSAID, Aspirin, cox-II inhibitor, ACE inhibitors, loop diuretics, antidepressants, self-rated health status, BMI, PASE score, walking speed

^e^CVD risk factors included systolic blood pressure, fasting blood glucose, total cholesterol and HDL

## Discussion

In the present study men with the highest levels of IL-6, TNF-α, or CRP had a higher odds of prevalent PAD compared to men with the lowest levels. Higher inflammation was associated with slower walking speed as well as a higher prevalence of hypertension, diabetes mellitus, COPD, CHF and higher fasting blood glucose. Nevertheless, the association between inflammation and PAD was independent of these risk factors. Smoking is a well-established risk factor for PAD and was also associated with greater inflammation. Adjustment for smoking attenuated the relationship between CRP and PAD, suggesting that smoking may influence risk of PAD through inflammatory pathways. Conversely, the association between IL-6 and TNFα and PAD remained statistically significant even after adjusting for smoking, supporting previous studies suggesting that inflammation itself is an independent risk factor for PAD [9, 10, 12–14, 24]. In our study, the magnitude of the association between highest inflammatory burden and PAD was similar to the increased odds of PAD associated with being 10 years older.

Men with PAD tended to have higher levels of every pro-inflammatory cytokine except IL-6 SR. Current literature supports that the strongest positive associations between PAD and cytokines are found with IL-6 [9, 13, 15, 25, 26] and CRP [9, 11, 13, 15, 26, 27] although there is some evidence for IL-6SR [9], TNFαSRI/II [15, 28] and a number of cytokines we did not measure in our study [9, 13, 29]. Our finding that TNFα was positively and significantly related with PAD has some strong support [26], although certain studies lack statistical significance [15] and others have actually reported the opposite [9]. Although previous studies have found conflicting results [9, 10, 30] we found a positive relationship between IL-10 and PAD in unadjusted analyses, which may represent a mechanism of overall inflammatory dysregulation in the disease. We found no association between IL-6SR and PAD, and the positive association between TNFαSRI/II with PAD was attenuated after multivariate adjustment. Nevertheless, our metric of inflammatory burden included all of the pro-inflammatory markers. Men with the greatest inflammatory burden had a >3-fold increased odds of PAD, but this was slightly attenuated after adjusting for smoking. These results suggest that overall inflammatory burden is related to PAD; however, smoking at least partially mediates this association.

Pro-inflammatory cytokines are implicated in other disease processes but the exact mechanism of their pathophysiology remains unclear. If particular inflammatory profiles are specific to one disease process over another (i.e. PAD versus CVD) it could lead to new screening tools, parameters for risk stratification, and targeted therapies. With an aging population and large anticipated influx of new “health care utilizers”, it is of paramount importance that screening tests be developed and exploited to curb morbidity and mortality from PAD and other diseases. Development of a screening cytokine panel to identify patients at risk of multiple diseases would have an important public health impact.

There are a number of strengths to our study. Our study consists of a population-based cohort and we measured seven cytokines with well-established, repeatable assays. Our use of ABI as a proxy for PAD is widely accepted [6] and we accounted for many possible confounders, including standard cardiovascular risk factors, physical activity, comorbidities and medications. Previous literature on PAD and inflammation often used a case–control approach or measured only a small number of cytokines.

Limitations of our study include the cross-sectional design. Our study population consisted of primarily Caucasian men and may not be generalizable to non-white men and women. We developed an inflammatory burden score by calculating the number of pro-inflammatory cytokines in the highest quartile. However, this approach yielded an uneven number of men in each group. For example, the number of men with 2 high inflammatory cytokines was much smaller than the other groups and CI were wide. Finally, we had no information on clinical signs and symptoms of PAD.

## Conclusions

In conclusion, individual pro-inflammatory cytokines and overall inflammatory burden are associated with prevalent PAD, an association similar to aging 10 years. The inflammatory effects of smoking at least partially mediates this association. To our knowledge we are the first to relate inflammatory burden to prevalent PAD, which could be expanded upon in the future as more cytokines/biomarkers are discovered, and the assays become faster, more sensitive, and less expensive. Future studies on incident PAD in the setting of various inflammatory profiles may allow us predict disease and intervene before there is significant morbidity.

## Abbreviations

ABI: ankle-arm index
CHF: congestive heart failure
CI: confidence interval
COPD: chronic obstructive pulmonary disease
CRP: C-reactive protein
CVs: coefficients of variability
FBG: fasting blood glucose
IDIS: Iowa Drug Information Service
IL-10: interleukin-10
IL-6SR: IL-6 soluble receptor
LCBR: Laboratory for Cytokine Biochemistry
MrOS: Osteoporotic Fractures in Men Study
MV: multivariable
OR: odds ratio
PAD: peripheral arterial disease
PASE: Physical Activity Scale for the Elderly
TNFα: tumor-necrosis factor-α
TNFαSRII: soluble receptor-II
